# Supplementary material for: Evaluation of Different Capture Solutions for Ammonia Recovery in Suspended Gas Permeable Membrane Systems
Source: Membranes (Basel). 2022 May 31;12(6):572. doi: 10.3390/membranes12060572 (PMC9228927; doi:10.3390/membranes12060572)
Supplement: Supplementary file 1 [file membranes-12-00572-s001.zip › membranes-1737694-supplementary.pdf]

Evaluation of different adsorbents for ammonia recovery in suspended gas permeable membrane systems

M. Soto-Herranz, M. Sánchez-Báscones, J.M. Antolín-Rodríguez and P. Martín-Ramos

<u>SUPPLEMENTARY MATERIALS</u>

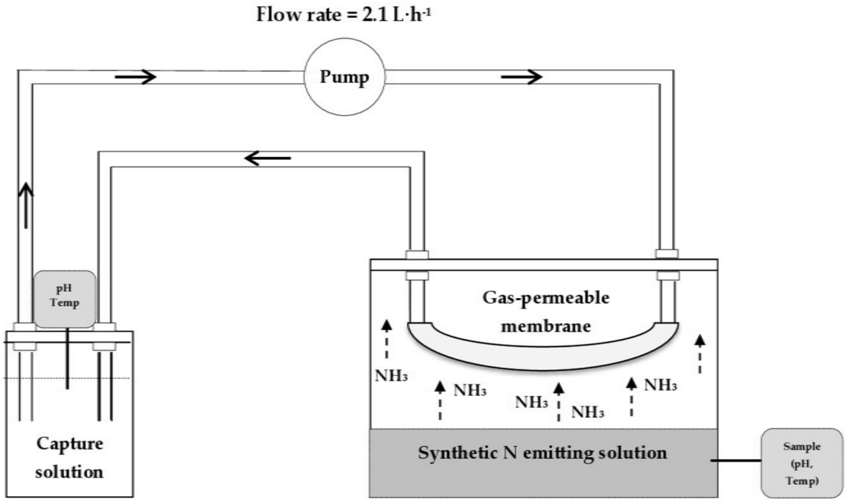

Figure S1. Scheme of the NH_3 capture process by the suspended gas-permeable membrane system in a closed circuit using different trapping solutions at 25 °C. Reproduced from [Membranes, 2021, 11(7):538; DOI: 10.3390/membranes11070538] under CC BY 4.0 license.

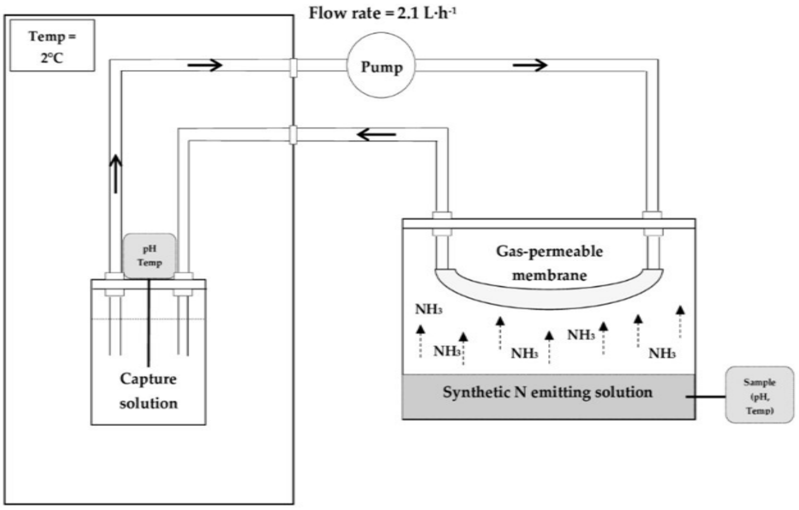

Figure S2. Scheme of the NH_3 capture process by the suspended gas-permeable membrane system in a closed circuit using different trapping solutions at 2 °C. Adapted from [Membranes, 2021, 11(7):538; DOI: 10.3390/membranes11070538] under CC BY 4.0 license.

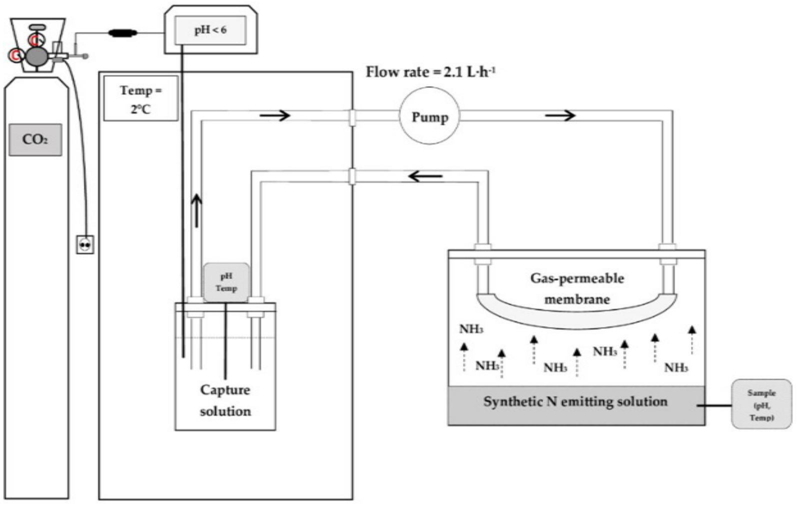

Figure S3. Scheme of the NH_3 capture process by the suspended gas-permeable membrane system in a closed circuit using carbonic acid as the ammonia trapping solution at 2 °C. Reproduced from [Membranes, 2021, 11(7):538; DOI: 10.3390/membranes11070538] under CC BY 4.0 license.

Table S1. Characteristics of the e-PTFE membrane used in the experiments.

Length (cm)	100
Outer diameter (mm)	5.2
Width of the wall (mm)	0.64
Polymer density (g/cm^3)	0.95
Porosity (%)	< 60
Average pore size length (μm)*	12.7 ± 5.9
Average pore size width (μm)*	1.3 ± 0.9
Absorption surface (cm^2)	163.4

* Pore sizes are average values across 10 membrane samples characterized by Scanning Electron Microscopy (SEM).

Table S2. Summary of operation and chemical costs, as well as end-product market prices, for the eight ammonia trapping solutions under analysis.

**Operation costs**	
Pumpdrive 5001 peristaltic pump	
Electrical consumption (kWh)	0.1
Cost (€/kWh)	0.3
Weekly electrical consumption (kW/7d)	16.8
Weekly cost (€/7d)	5.0
**Chemical costs**	
Acid consumption	
Sulphuric acid (g)	51.3
Phosphoric acid (g)	38.3
Nitric acid (g)	96.6
Carbonic acid (g)	59.1
Acetic acid (g)	60.1
Citric acid (g)	64.0
Maleic acid (g)	58.6
Acid cost	
Sulphuric acid (€/L) / (€/kg)	47.6 / 87.1
Phosphoric acid (€/L) / (€/kg)	44.9 / 80.4
Nitric acid (€/L) / (€/kg)	37.4 / 52.0
Carbonic acid (€/L) / (€/kg)	- / 89.5
Acetic acid (€/L) / (€/kg)	46.4 / 48.7
Citric acid (€/kg)	- / 81.2
Maleic acid (€/Kg)	- / 105.8
**Fertilizer bulk prices**	
Ammonium sulphate 21% (€/kg)	0.69 [1]
Diammonium Phosphate (DAP) 18-46-0 (€/kg)	1.09 [1]
Ammonium nitrate 34,5% (€/kg)	1.08 [2]
Ammonium bicarbonate 99-100% (€/kg)	0.93 [3]
Ammonium acetate 99% (€/kg)	1.11 [3]
Ammonium citrate > 90% (€/kg)	1.06 [3]
Ammonium maleate (€/Kg)	2.76 [4]
$NH_3 \cdot H_2O$ 25% (€/kg)	1.30 [3]

The prices of the end products have been obtained from the suppliers listed below:

1 North Carolina Dept of Ag-USDA Market News Service, 2022. Available online at: https://mymarketnews.ams.usda.gov/filerepo/sites/default/files/3159/2022-04-14/579600/ams_3159_00112.txt (accessed 14 April 2022).

2 Almacenes Antonio Guerrero. Agricultura y Ganadería-Convencional y Ecológica, 2022. Available online at: https://www.almacenesantonioguerrero.es/productos/nitrato-345-25kg/%0A.Best-price-In-stock/2051660.html (accessed 16 April 2022).

3 Various suppliers, 2022. Available online: https://www.made-in-china.com/ (accessed 14 April 2022).

4 Hangzhou LookChem Network Technology Co. Ltd. Available online at: https://www.lookchem.com/product_Ammonium-hydrogen-maleate-Manufacturer-High-quality-Best-price-In-stock/2051660.html (accessed 16 April 2022).
